# Characterization of occult hepatitis B virus infection among HIV positive patients in Cameroon

**DOI:** 10.1186/s12981-017-0136-0

**Published:** 2017-03-08

**Authors:** George Gachara, Tshifhiwa Magoro, Lufuno Mavhandu, Emmaculate Lum, Helen K. Kimbi, Roland N. Ndip, Pascal O. Bessong

**Affiliations:** 1 HIV/AIDS & Global Health Research Program, Department of Microbiology, University of Venda, Private Bag X5050, Thohoyandou, 0950 Limpopo South Africa; 2 Department of Medical Laboratory Sciences, Kenyatta University, Nairobi, Kenya; 3 Department of Zoology and Animal Physiology, Faculty of Science, University of Buea, Buea, Cameroon; 4 Department of Biological Sciences, Higher Teachers’ Training College, University of Yaounde, Yaounde, Cameroon; 5 Department of Medical Laboratory Science, Faculty of Health Sciences, University of Bamenda, Bambili, Bamenda, Cameroon; 6 Department of Biochemistry and Microbiology, Faculty of Science and Agriculture, University of Fort Hare, Alice, South Africa; 7 Department of Microbiology and Parasitology, University of Buea, Buea, Cameroon

**Keywords:** Hepatitis B virus, Occult hepatitis B infection, HIV, Cameroon

## Abstract

**Purpose:**

Occult hepatitis B infection (OBI) among HIV positive patients varies widely in different geographic regions. We undertook a study to determine the prevalence of occult hepatitis B infection among HIV infected individuals visiting a health facility in South West Cameroon and characterized occult HBV strains based on sequence analyses.

**Methods:**

Plasma samples (n = 337), which previously tested negative for hepatitis B surface antigen (HBsAg), were screened for antibodies against hepatitis B core (anti-HBc) and surface (anti-HBs) antigens followed by DNA extraction. A 366 bp region covering the overlapping surface/polymerase gene of HBV was then amplified in a nested PCR and the amplicons sequenced using Sanger sequencing. The resulting sequences were then analyzed for genotypes and for escape and drug resistance mutations.

**Results:**

Twenty samples were HBV DNA positive and were classified as OBI giving a prevalence of 5.9%. Out of these, 9 (45%) were anti-HBs positive, while 10 (52.6%) were anti-HBc positive. Additionally, 2 had dual anti-HBs and anti-HBc reactivity, while 6 had no detectable HBV antibodies. Out of the ten samples that were successfully sequenced, nine were classified as genotype E and one as genotype A. Three sequences possessed mutations associated with lamivudine resistance. We detected a number of mutations within the major hydrophilic region of the surface gene where most immune escape mutations occur.

**Conclusions:**

Findings from this study show the presence of hepatitis B in patients without any of the HBV serological markers. Further prospective studies are required to determine the risk factors and markers of OBI.

**Electronic supplementary material:**

The online version of this article (doi:10.1186/s12981-017-0136-0) contains supplementary material, which is available to authorized users.

## Background

Hepatitis B, a potentially life-threatening liver infection caused by the hepatitis B virus (HBV) is a major global health problem. Of the two billion people infected with the virus, more than 240 million are chronic carriers [1], and more than 686,000 die annually from HBV-related complications, including cirrhosis and hepatocellular carcinoma [2]. A growing body of evidence is emerging showing that the prevalence of HBV is significantly higher amongst HIV-positive individuals, presumably because of the shared transmission risks and risk factors [3, 4]. HIV generally accelerates the natural course of HBV infection and facilitates faster progression of liver disease to cirrhosis and hepatocellular carcinoma (HCC) [5].

Traditionally, HBV is diagnosed by serological techniques to detect antigens or antibodies. The hepatitis B surface antigen (HBsAg) is often used for routine diagnosis since it is considered as the hallmark of infection. During acute infection, antibodies to HBV core antigens (anti-HBc) (initially both IgM and IgG) appear 1–2 weeks after the appearance of HBsAg, while IgG persists during chronic infection. The presence of antibodies to HBsAg (anti-HBs) represents immunity to HBV infection [6].

In the advent of molecular diagnostics, it has been shown that a number of individuals may harbour HBV-DNA at very low levels in their liver and/or serum despite the absence of detectable HBsAg by currently available assays [7]. This is termed occult hepatitis B infection (OBI), characterized by the presence of HBV DNA in the blood and liver in HBsAg-negative individuals, who may or may not have anti-HBc and anti-HBs [6]. There are several mechanisms that have been hypothesized to lead to development of OBI. These include development of HBV S gene mutants that affect the detectability of the virus by conventional HBsAg assays, strong suppression of viral replication and reduced expression of HBsAg, epigenetic mechanisms and co-infection with other viruses [8].

OBI is frequent in persons with HIV, and its prevalence varies considerably in different geographic regions [9]. It has been shown that immunosuppression due to HIV infection could lead to low antibody response to HBsAg and also HBV reactivation [10, 11]. Consequently, among HIV patients who test HBsAg negative, HBV DNA should be determined before starting highly active antiretroviral therapy (HAART) so that anti-HBV antiretrovirals can be included if necessary. The prevalence of OBI however, has been shown to vary in different demographic settings. A recent study in Cameroon reporting on OBI focused on patients from a health centre in the capital Yaoundé [12]. It would be expected therefore that the two regions of Cameroon from where the patients in the current study were derived would provide added information on OBI in this country where both HBV and HIV are endemic.

## Methods

### Study design and setting

This was a cross-sectional retrospective study using 337 HBsAg negative plasma samples from 455 HIV positive outpatient clients of the Mutengene Baptist Health Centre located in the South West Region of Cameroon. The health centre offers HIV/AIDS treatment, prevention, medical, spiritual and psychosocial care in the Tiko Health District. The hospital attends to at least 8000 patients per month. Patients come from various towns such as Buea, Limbe, Tiko, Kumba in the South West Region, and Douala, Nkongsamba, in the Littoral Region. Details of the study setting have been reported [13].

### Sample collection and processing

All consenting participants completed a questionnaire regarding their socio-demographic characteristics. Five millilitres of venous blood was collected once from each consenting HIV positive adult individual using EDTA vacutainer tubes while two ml of blood was collected from young children (<5 years). Blood was centrifuged for 5 min at 3578×*g*. Following centrifugation, plasma was aspirated aseptically and aliquoted into sterile labelled cryotubes and stored at −80 °C. These were then transported on dry ice to the HIV/AIDS & Global Health Research laboratories at the University of Venda, South Africa where they were stored at −80 °C until used.

### Serological testing

Initially, all samples were screened for HBsAg using a commercially available direct enzyme-linked immunosorbent assay (ELISA) kit (Bioelisa, Biokit, Barcelona, Spain). In this study, samples that were HBsAg negative were further screened for anti-HBc and anti-HBs using another ELISA commercial kit (DRG Instruments GmbH, Germany).

### DNA extraction

DNA from HBsAg negative samples was extracted from 100 µl of plasma using the Quick-gDNA mini prep kit (Zymo research, USA) according to the manufacturer’s instructions. The extracted DNA was amplified immediately after extraction or stored at −20 °C awaiting subsequent amplification.

### Polymerase chain reaction (PCR) for HBV surface/polymerase gene

A nested PCR was performed in order to amplify the overlapping surface/polymerase gene covering nucleotides 403–768 from the EcoR1 site using primers and protocols described previously [14]. This generates a 366 bp product (Additional file 1: Figure S1). The first round reaction was conducted in a 50 µl volume containing 10 mM Tris-HCI pH 8.3, 50 mM potassium chloride, 0.2 mM dNTP mix, 2.5 mM magnesium chloride, 0.2 ng/µl of each primer, and 2 units of *Taq* polymerase (Applied Biosystems, PE, Italia). The thermocycling conditions involved 35 cycles of denaturation at 95 °C for 1 min, annealing at 55 °C for 1 min, and extension at 72 °C for 1 min. Five microlitre of the first round PCR product was used as a template for the nested PCR under the same reaction conditions, but performing only 20 cycles. The PCR products were then resolved by 1.5% agarose gel electrophoresis stained with ethidium bromide. PCR amplicons were then purified using the QiAquick PCR Purification Kit (Qiagen, Hilden; Germany) according to the manufacturer’s instructions.

### Sequencing and sequence analysis

Purified PCR amplicons were directly sequenced at Inqaba Biotech (Pretoria, South Africa) according to the Sanger protocol. Contiguous nucleotide sequences (contigs) were assembled from resulting forward and reverse reactions using the SeqMan Pro^®^ module of the Lasergene (version 8.1.5) sequence analysis software suite (DNASTAR. Madison, WI.).

The resulting nucleotide sequences were aligned using the Clustal W program implemented in MEGA 6.06 [15]. They were also translated and checked for HBsAg mutations in the S gene and drug resistance associated mutations in the P gene. Phylogenetic reconstruction was carried out using the MrBayes program version 3.1.2 [16]. The Bayesian tree was inferred by running a Markov-chain Monte Carlo (MCMC) algorithm for 1 million generations, sampling at every 100th generation with a burn in setting of 10% of generations. The GTR+G+I model (general time-reversible model with gamma distributed rates of variation among sites and a proportion of invariable sites) was found to be the best-fit model for our dataset. The resulting phylogenetic tree was visualized using FigTree, version 1.3.1 (http://tree.bio.ed.ac.uk/software/figtree/) and used to determine the HBV genotype.

### Statistical analysis

A database was prepared and processed using SPSS 20 (IBM, Chicago, IL, USA). Fisher’s exact test was used to analyze the socio-demographic and clinical variables associated with OBI. A p value of less than 0.05 was considered statistically significant.

## Results

### Prevalence of occult HBV infection

HBV DNA was detected in 20 out of the 337 HBsAg negative samples giving an OBI prevalence of 5.9% in this study. OBI was higher among females, the unemployed, patients with low hemoglobin, those with normal white blood cell count and malaria negative individuals. However none of these variables were statistically associated with OBI (Table 1).

**Table 1:** The prevalence of occult hepatitis B infection (OBI), demographic variables, and associated risk factors among HIV/HBV infected patients in Cameroon

Variable	OBI negative (%)	OBI positive (%)	p value
Gender			0.303
Male	85 (26.8)	3 (15.0)	
Female	232 (73.2)	17 (85.0)	
Age-group (years)			0.382
<15	30 (9.5)	4 (20.0)	
15–24	14 (4.4)	0 (0.0)	
25–35	140 (44.2)	6 (30.0)	
36–45	85 (26.8)	6 (30.0)	
>45	48 (15.1)	4 (20.0)	
Employment status			0.776
Employed	67 (21.1)	3 (15.0)	
Unemployed	250 (78.9)	17 (85.0)	
Malaria infection			1.00
Malaria positive	107 (33.8)	7 (35.0)	
Malaria negative	210 (66.2)	13 (65.0)	
WHO clinical stage			0.179
Stage 1	67 (21.7)	3 (15.0)	
Stage 2	87 (28.1)	7 (35.0)	
Stage 3	100 (32.4)	7 (35.0)	
Stage 4	55 (17.8)	3 (15.0)	
CD4 count			1.00
<350	174 (54.9)	11 (45.0)	
>350	143 (45.1)	9 (55.0)	
Haemoglobin level			1.00
Low Hb	220 (69.4)	14 (70.0)	
Normal Hb	97 (30.6)	6 (30.0)	
White blood cell count			0.193
Low WBC count	82 (25.9)	8 (40.0)	
Normal WBC count	235 (74.1)	12 (60.0)	

### Serological profile of occult HBV infection

All the study samples were assayed for anti-HBs and anti-HBc serological markers. Out of the OBI positive samples, 9 (45%) were anti-HBs positive while 10 (52.6%) were anti-HBc positive (Table 2). Additionally, 2 individuals had dual anti-HBs and anti-HBc reactivity while 6 had no detectable HBV antibodies.

**Table 2:** Serological characterization of OBI individuals among HIV/HBV infected patients in Cameroon

OBI status	Anti-HBs status^a^		Anti-HBc status^b^	
	Anti-HBs^+^	Anti-HBs^−^	Anti-HBc^+^	Anti-HBc^−^
OBI positive^c^	5	14	10	9
OBI negative	104	201	176	134

^a^This was done for 324 patients due to insufficient sample volume

^b^This was done for 329 patients due to insufficient sample volume

^c^There was insufficient sample volume to conduct both anti-HBs and anti-HBc screening for one patient who was OBI positive

### Molecular and genetic characterization of occult HBV DNA

**Fig. 1:**
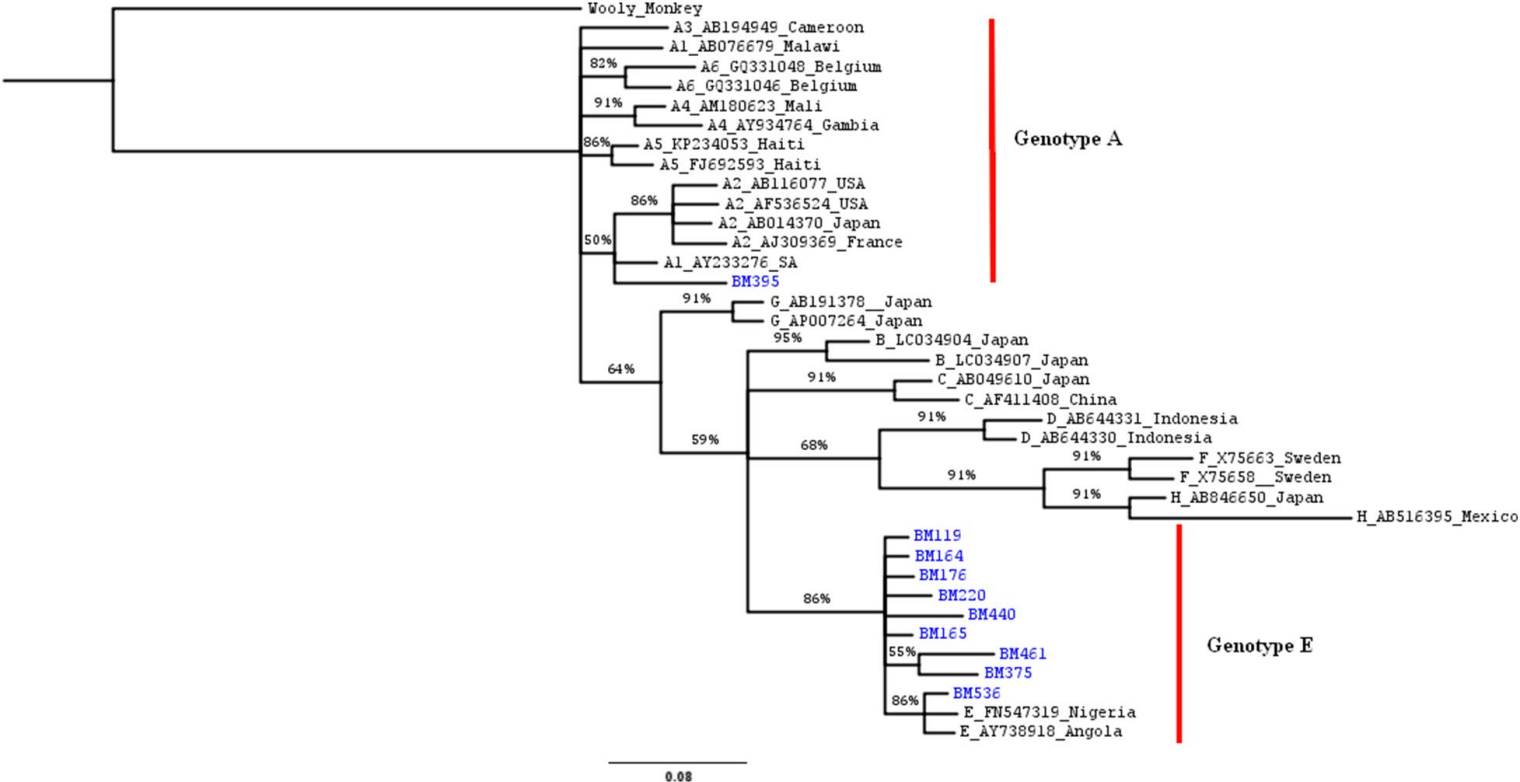
A bayesian rooted phylogenetic tree constructed using MrBayesver 3.1.2 of the Cameroon OBI sequences from HBV/HIV co-infected individuals. Twenty-eight global sequences obtained from GenBank were included to support tree topology and genotype identification. The ten viruses characterised in this study are named with a prefix BM and highlighted in *blue* at the taxa

Sequencing of the overlapping surface/polymerase gene was done for 10 out of the 20 OBI positive DNA samples. Phylogenetic analyses indicated that HBV genotype E and A occurred in 9 (90%) and 1 (10%) individuals respectively (Fig. 1).

**Fig. 2:**
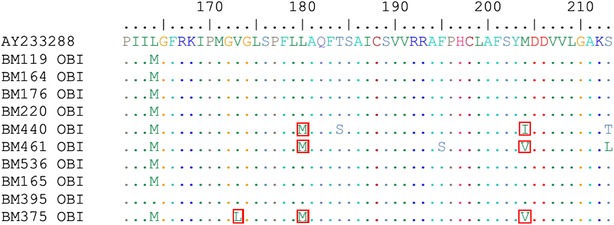
Amino acid alignment of the RT domain (aa 161–213) of the P gene of HBV/HIV co-infected individuals from Cameroon. The Cameroon samples are named with a prefix BM and the drug resistance conferring mutations V173L, L180M, and M204V/I are shown. The numbering on* top* indicates the amino acid position

Mutations in the “*a”* determinant within the major hydrophilic region (MHR) of the S gene may cause a conformational change in HBsAg, leading to undetectability of HBsAg (diagnostic-escape) as well as evading the host’s immune response (immune-escape). The main mutation observed in this region was the G159A mutation observed in eight (80%) of the samples compared to 41.3% among HBsAg positive samples previously observed. Also, nine of the samples possessed leucine at amino acid 127. Examination of the RT domain of the polymerase gene indicated that three of the sequences had mutations associated with drug resistance. These mutations V173L, L180M, T184S and M204V/I are associated with resistance to lamivudine, entecavir and telbivudine (Fig. 2). The three patients harboring these mutations had treatment experience with Lamivudine as part of their antiretroviral therapy.

## Discussion

Laboratory detection of Hepatitis B virus infection is crucial for global control and prevention of HBV disease. Among HIV infected individuals under HAART, the increased longevity may facilitate emergence of chronic liver disease which is often a cause of increased morbidity and mortality. A significant proportion of this burden may be attributed to occult hepatitis B virus infection since it has been shown to have hepatopathogenic potential. Furthermore, HAART creates immune reconstitution that can result in immune mediated liver injury and elevation of liver enzymes [17]. This may easily be misclassified as HAART-associated liver toxicity.

In this study, we have found a 5.9% prevalence of OBI among a group of HIV positive individuals in Cameroon and no significant association between the studied demographic parameters and risk factors of the participants and OBI was found.

The prevalence of OBI in this study is similar to the 5.9% found among HIV individuals in Sicily, Italy [18]. However, the observed prevalence is slightly lower than the 6.9 and 9.8% reported in Yaoundé, Cameroon and Khartoum, Sudan respectively [12, 19]. It should be noted however that the prevalence of OBI is dependent on the sensitivity of the DNA assay used, demography and the population studied [7]. Thus, the Sudan study used a more sensitive real time PCR method compared to the conventional PCR method used in this study. We propose that the difference in prevalence with the Yaoundé study may be due to demographics. Among HIV patients, several studies conducted worldwide have reported prevalence of OBI ranging from 0% to more than 90% [20, 21].

The current study did not find any association between OBI and the studied variables. This finding has also been made among Cuban HIV patients [22]. Generally, previous studies have made conflicting findings regarding factors associated with OBI. For example, Stuart et al. [20] have reported a significant association between low CD4 and OBI among HIV patients while other studies have not made this finding [22, 23]. Perhaps this may be due to the different diagnostic algorithms used to define OBI in the various studies.

Slightly more than half (52.6%) of the OBI positive individuals were anti-HBc positive, while 45% were anti-HBs positive. Our findings on the anti-HBc status is consistent with several studies that indicate the role of the anti-HBc profile as the most common serological surrogate of OBI although higher prevalence of anti-HBc alone does not necessarily reflect significantly higher frequency of OBI [24, 25]. Dual anti-HBc and anti-HBs reactivity was observed in 2 (20%) of the samples. Additionally, we identified six OBI positive individuals with no serologic evidence of infection (negative for HBsAg, anti-HBc, and anti-HBs). This is an indication that occult HBV can occur either in patients with serological evidence of past “apparently resolved” HBV infection, or also in individuals with no evident history of exposure to HBV [25]. Indeed this finding has epidemiological implications in that the burden of HBV in Cameroon, often determined by serological assays, is significantly underestimated. This ‘silent’ infection also has clinical implications since it leads to an increased risk of hepatocellular carcinoma [26].

The ten HBV strains sequenced in this study showed the circulation of both genotype E and A with genotype E predominating. Our findings are in agreement with previous studies in Cameroon that have documented the circulation of these two genotypes [12, 27]. Drug resistant patterns indicate that three of the strains had mutations associated with resistance to lamivudine, telbivudine and entecavir. Further, that the individuals harboring viruses with these mutations were all lamivudine experienced. Importantly though, none of the viruses harbored mutations associated with tenofovir and adefovir resistance. This finding is in agreement with the recommendation to include tenofovir as part of HAART among HIV/HBV co-infected patients [28]. Regarding mutations in the “*a”* determinant of the S gene, majority of the viruses had a G159A mutation. In addition, all the viruses had 127Lin this region. The 127L mutation has previously been associated with OBI [29], while the role of G159A in OBI is unclear.

The findings in this study should be interpreted in the light of several limitations. First, the HBV viral loads which are also often used as a marker of OBI were not available. Secondly, the study utilized a qualitative and not quantitative ELISA format, thus antibody titres were not determined. Thirdly, owing to resource constraints only 50% of the OBI positive samples were sequenced and lastly, the lack of liver enzyme levels precludes our ability to correlate the results reported here with clinical outcomes.

## Conclusion

In conclusion, we report a 5.9% prevalence of OBI among HIV infected individuals in Cameroon and the dominance of HBV genotype E. We also show the occurrence of OBI in the absence of any serologic evidence of infection; and the presence of mutations associated with lamivudine drug resistance in the study cohort. Further prospective studies in a larger cohort are needed to determine the clinical implications of OBI in this region.

## Additional file

**Additional file 1: Figure S1.** A representation gel showing amplified HBV DNA from HBsAg negative plasma. Lane L is a 100 bp molecular weight marker. Expected DNA band sizes of 366 bp were readily detected for samples on lanes 2, 5, 7, 8, 10–14. Lane P is a positive control obtained from the PCR optimization process and confirmed by sequencing. Lane N is a negative control (sterilized distilled water used as template).
