# Supplementary material for: Characterization of occult hepatitis B virus infection among HIV positive patients in Cameroon
Source: AIDS Res Ther. 2017 Mar 8;14:11. doi: 10.1186/s12981-017-0136-0 (PMC5341455; doi:10.1186/s12981-017-0136-0)
Supplement: Supplementary file 1 — Additional file 1: Figure S1. A representation gel showing amplified HBV DNA from HBsAg negative plasma. Lane L is a 100 bp molecular weight marker. Expected DNA band sizes of 366 bp were readily detected for samples on lanes 2, 5, 7, 8, 10–14. Lane P is a positive control obtained from the PCR optimization process and confirmed by sequencing. Lane N is a negative control (sterilized distilled water used as template). [file 12981_2017_136_MOESM1_ESM.docx]

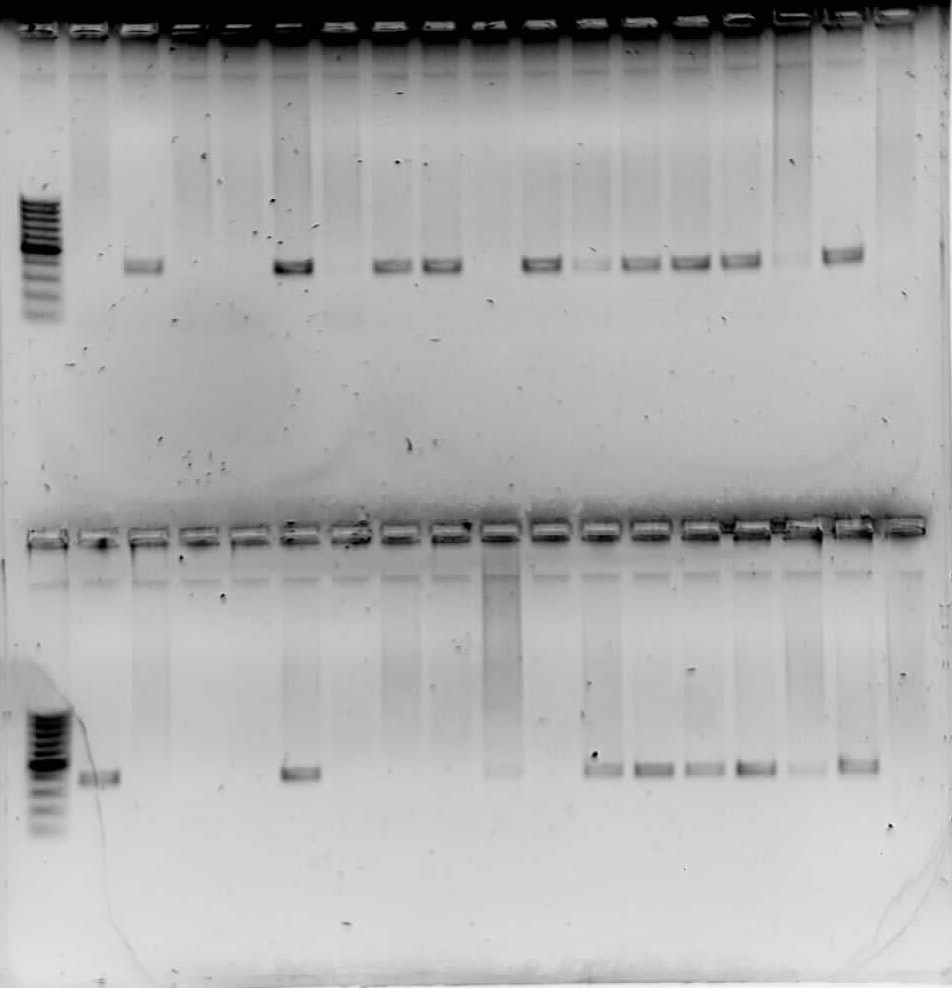


L 1 2 3 4 5 6 7 8 9 10 11 12 13 14 15 P N

**Figure S1**: A representation gel showing amplified HBV DNA from HBsAg negative plasma. Lane L is a 100 bp molecular weight marker. Expected DNA band sizes of 366bp were readily detected for samples on lanes 2, 5, 7, 8, 10-14. Lane P is a positive control obtained from the PCR optimization process and confirmed by sequencing. Lane N is a negative control (sterilized distilled water used as template).
